# Processing Laue Microdiffraction Raster Scanning Patterns with Machine Learning Algorithms: A Case Study with a Fatigued Polycrystalline Sample

**DOI:** 10.3390/ma15041502

**Published:** 2022-02-17

**Authors:** Peng Rong, Fengguo Zhang, Qing Yang, Han Chen, Qiwei Shi, Shengyi Zhong, Zhe Chen, Haowei Wang

**Affiliations:** 1 Chengdu Aircraft Industrial (Group) Co., Ltd., Chengdu 610073, China; rongpeng-love@163.com; 2 State Key Laboratory of Metal Matrix Composites, Shanghai Jiao Tong University, Shanghai 200240, China; sjtuheyq@sjtu.edu.cn (Q.Y.); sjtuch23@sjtu.edu.cn (H.C.); sqw@sjtu.edu.cn (Q.S.); shengyi.zhong@sjtu.edu.cn (S.Z.); zhe.chen@sjtu.edu.cn (Z.C.); hwwang@sjtu.edu.cn (H.W.); 3 Anhui Province Engineering Research Center of Aluminium Matrix Composites, Huaibei 235000, China; 4 SJTU-ParisTech Elite Institute of Technology, Shanghai Jiao Tong University, Shanghai 200240, China

**Keywords:** Laue microdiffraction, unsupervised machine learning, fatigued microstructure

## Abstract

The massive amount of diffraction images collected in a raster scan of Laue microdiffraction calls for a fast treatment with little if any human intervention. The conventional method that has to index diffraction patterns one-by-one is laborious and can hardly give real-time feedback. In this work, a data mining protocol based on unsupervised machine learning algorithm was proposed to have a fast segmentation of the scanning grid from the diffraction patterns without indexation. The sole parameter that had to be set was the so-called “distance threshold” that determined the number of segments. A statistics-oriented criterion was proposed to set the “distance threshold”. The protocol was applied to the scanning images of a fatigued polycrystalline sample and identified several regions that deserved further study with, for instance, differential aperture X-ray microscopy. The proposed data mining protocol is promising to help economize the limited beamtime.

## 1. Introduction

Laue diffraction, that may occur when a polychromatic X-ray beam illuminated a crystal, was first discovered in 1912, and has unveiled both the electromagnetic nature of X-ray and the periodic ordering of atoms in crystal [1]. Thanks to the polychromaticity of the employed X-ray, multiple diffraction peaks can be recorded in a single exposure without any rotation, thereby excluding the ambiguity of the illuminated volume [2]. With the development of polychromatic beam focusing optics, notably Kirkpatrick–Baez mirrors, micron-sized high-brilliance polychromatic X-ray beam can be produced at synchrotron radiation sources and directed to probe inside materials with submicrometric spatial resolutions, i.e., Laue microdiffraction [3]. Compared to the electron backscatter diffraction (EBSD) technique, Laue microdiffraction technique functions by raster scanning the sample to generate the lattice orientation and distortion maps from the one-by-one analysis of the diffraction pattern emanating from each scanned spot [4,5,6]. The two techniques are comparable [7] and complementary to each other [8]. It is generally accepted that EBSD has an edge on finer spatial resolution of nanoscale, whilst Laue microdiffraction can have a much better accuracy on the lattice orientation and distortion with an attainable order of 10^−9^ [9].

A salient feature of Laue microdiffraction is its sensitivity to the local misorientation inside the illuminated volume [10,11], more specifically, the fragmentation of Laue spot may indicate the presence of geometrically necessary boundaries (GNBs) and the elongation of Laue spot discloses the presence of geometrically necessary dislocations (GNDs). Although a critical aspect of the spot shape analysis lies on the assumption that the dislocations were dominantly edge-type in the illuminated volume, a recent study with focused ion beam and transmission electron microscopy confirmed that this analysis stood still if the dislocations had predominately screw-type [12]. With the aid of a wire profiler (typically Pt), the shape of spot can be spatially resolved to yield a subsurface, 3D mapping of lattice orientation and distortion non-destructively [13,14,15], namely the differential-aperture X-ray microscopy (DAXM) technique [16].

The rotation-free feature of Laue microdiffraction renders it suitable for in-situ or ex-situ mechanical experiments [17,18,19,20,21,22]. Nevertheless, considerable efforts have to be made to ensure the consistency of the illuminated volumes at the moments of image acquisition, e.g., either using the fiducial markers deposited to the sample surface [23], or resorting to the digital image correlation (DIC) code [24]. Special attention needs also to be paid to the instability of experimental setup due to mechanical loading as well as the image noise [25,26,27].

Despite the wealth of information behind Laue microdiffraction pattern, the interpretation is not straightforward since the wavelength or index pertaining to each diffraction peak is not known a priori. Standard treatment involves modulating the orientation and calibration parameters to minimize the discrepancy between the simulated and experimental diffraction pattern, and has been implemented in software such as XMAS [28] and LaueTools (https://gitlab.esrf.fr/micha/lauetools, accessed on 16 February 2022). The standard treatment is in essence a trial-and-error process that usually suffers from inefficiency, especially for the raster scanned diffraction patterns which has to be treated one by one. Therefore, any additional information concerning the scanned microstructure would facilitate the process, for example, Örs et al. [8] used the orientation obtained by EBSD to overcome the difficulty in indexing the Laue microdiffraction patterns of low-symmetry crystals; Kou et al. [29] suggested indexing one Laue microdiffraction pattern per grain as the reference with which the rest patterns of the grain could be analyzed without indexation.

In a word, the indexation of diffraction peaks is the key to the full interpretation of diffraction pattern. Nevertheless, in certain circumstances, full interpretation of diffraction patterns is unnecessary or fast parallel computing capabilities are unavailable, thereby necessitating the development of indexation-free approach towards on-the-fly analysis of raster scanned diffraction patterns. Zhou et al. [30] proposed using the distribution of average recorded intensities and average filtered intensities of the raster scanned diffraction patterns to visualize the characteristics of microstructural features. Recent application of convolutional neural networks (CNN) to diffraction patterns has been involved with the extraction of features for further clustering and labeling the raster scanned diffraction patterns [31] or for the identification of crystal structures [32].

In the present work, we demonstrated the application of unsupervised machine learning algorithms to the raster scanning Laue microdiffraction images of the fatigued polycrystalline copper. Substantial dislocation structures will grow in copper after the cyclic loading [33], deteriorating the identifiability of the diffraction pattern. Although template matching schemes have been shown applicable to the indexation and misorientation analysis of smeared diffraction patterns [34], huge amount of calculation was still inevitable and the reliability of outcome would be degraded in line with the formation of dislocation structures. On the other hand, machine learning algorithms, which were developed to handle big data, were possible to circumvent the difficulty of indexation and cluster raster scanning diffraction images according to their features, thereby mapping phases, grains, or grain substructures. In this work, we designed a protocol to mine regions of interest from the massive amount of raster scanning diffraction images by using unsupervised machine learning algorithms. The protocol has been applied to the scanned diffraction images of a fatigued specimen and yielded results meaningful to a material scientist. The results were re-examined by checking the underlying diffraction images.

## 2. Experiment

**Figure 1:**
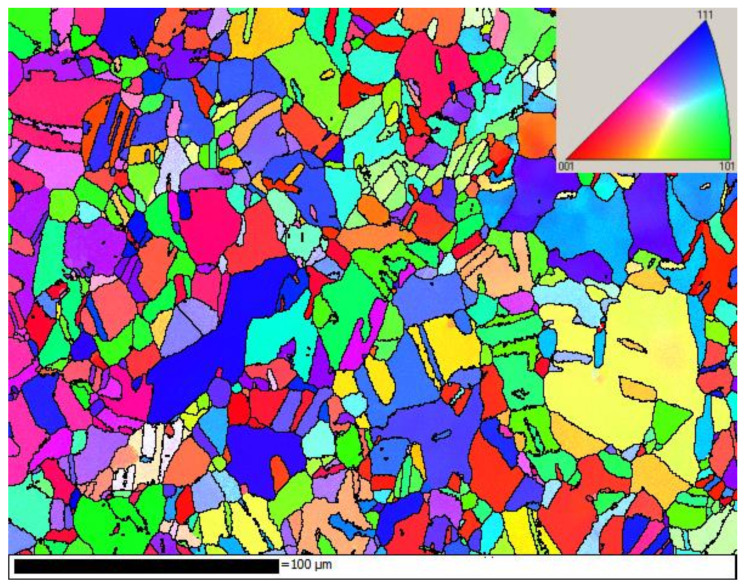
The grain structure of the sample colored by (IPF) with axis vertical to the grain structure map.

The diffraction images were collected from raster scanning of fatigued polycrystalline copper. (Huye Co., Ltd., Suzhou, China) The sample was designed in accordance with the ASTM/E606 standard. Figure 1 shows the grain structure of the sample colored by inverse pole figure (IPF) with axis vertical to the grain structure map. The sample, cyclically loaded in stress-control mode with the stress varying sinusoidally within the range 0 ~ 140 MPa, has undergone a maximum strain of ~ 10% in the initial cycle and cyclic creep in the subsequent cycles. The sample was fatigued up to 10^9^ cycles with a frequency of 10 Hz.

**Figure 2:**
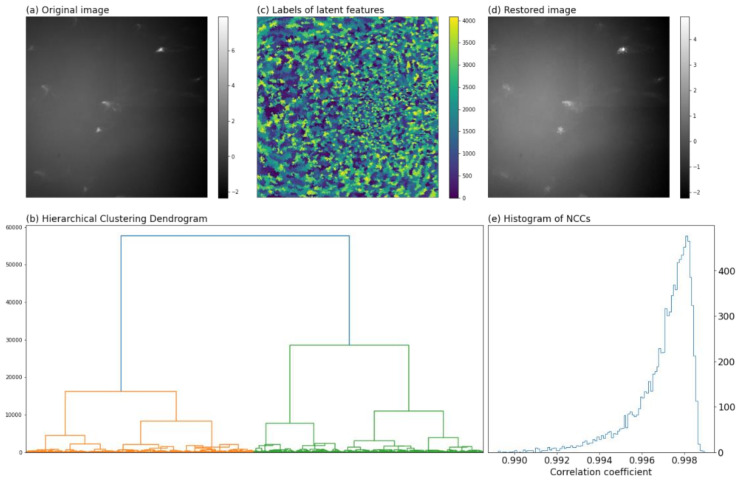
(**a**) One example of diffraction patterns after normalization and 2 × 2 averaging binning; (**b**) hierarchical clustering dendrogram of pixels for feature extraction; (**c**) the distribution of latent features colored by their labels; (**d**) image restored from the latent features; (**e**) histogram of NCCs.

The Laue raster scanning over the sample was performed in beamline 4B of Pohang Light Source (Pohang, South Korea). The raster scanning was over a V×H grid with the vertical direction parallel to the fatigue loading wherein V=64 and H=146. The step size was 2 μm in both the horizontal and vertical directions of the grid. The obtained Laue microdiffraction pattern was extremely blurred with almost no discernable diffraction peaks (Figure 2a).

## 3. Methodology

### 3.1. Feature Extraction

The original Laue microdiffraction pattern has 1024 × 1024 pixels. Although one can directly use the full gray levels of images to segment the grid points from a theoretical point of view, it is neither practical nor necessary to handle such huge amount of data, thus necessitating a feature extraction process to reduce the diffraction patterns into a manageable number of latent features. The extracted features would subsequently lay foundation for grid segmentation.

To begin with, each image needed to be normalized to eliminate systematic errors. Normalization was accomplished by subtracting the mean gray level from the gray level and dividing by the standard deviation of the gray levels, in accordance with McAuliffe et al. [35].

Then, since the background occupying the majority of Laue pattern contained nothing other than noise, a moderate resolution coarsening could speed up the image processing and smooth the noise [31]. In this regard, the normalized images were compressed to 512 × 512 pixels by 2 × 2 binning (averaging). Figure 2a showed one example of the resultant images.

A number of algorithms serve to extract features for further grid segmentation. Song et al. [31] applied CNN to extract latent features. However, at present, the authors did not have sufficient patterns to train the CNN, therefore unsupervised machine learning algorithms that did not require training dataset were employed herein to extract latent features of each pattern. Here we used the hierarchical agglomerative clustering (HAC) algorithm [36] backed by Scikit-Learn [37]. When treating the scanning diffraction patterns with HAC algorithm, each pixel corresponded to a vector comprised of the values at the pixel in whole diffraction patterns; then a metric (Euclidean distance, maximum distance, etc.) was used to quantify the dissimilarity between a pair of pixels; then pixels with high similarities were merged to form a feature according to a linkage criterion. The connectivity of pixels could be exploited to facilitate the merging process such that only the pairs of adjacent pixels were under consideration. In this work the Euclidean distance was used as the metric of dissimilarity, and the linkage criterion employed was “ward”, aiming at minimizing the sum of squared differences within all clusters.

Figure 2b visualized the merging process with a tree diagram called dendrogram: the leaves of the dendrogram corresponded to the pixels of the image; two leaves with the highest similarity were merged to form a branch in the first step; in the following steps, two branches with the highest similarity were fused into one branch until only one branch was left. The ordinate of Figure 2b gave the dissimilarities between two merging branches (or leaves). The branches that were mutually exclusive and collectively incorporated all leaves constituted the latent features of the images.

Conceivably, increasing the number of latent features $N_{f}$ led to better reproducibility of the original data, however at the expense of the data compression. We empirically set $N_{f}$ to be 4096 (the compression rate was $\frac{4096}{512^{2}}=1.5625\%$), with which the original image could be well restored as shown hereinafter. Figure 2c showed the distribution of features colored by the indices of latent features. Figure 2d showed the restored image of Figure 2a from its latent features, wherein high resemblance of the two images could be identified.

A quantitative metric of the resemblance between the original and restored image is the normalized cross-correlation (NCC) coefficient:

(1) $$\begin{array}{c} NCC≐\frac{\sum_{i}\sum_{j}I_{ij}^{\mathrm{original}}I_{ij}^{\mathrm{restored}}}{\sqrt{\sum_{i}\sum_{j}{\left(I_{ij}^{\mathrm{original}}\right)}^{2}\sum_{i}\sum_{j}{\left(I_{ij}^{\mathrm{restored}}\right)}^{2}}},\# \end{array}$$

where $I_{ij}^{\mathrm{original}}$ and $I_{ij}^{\mathrm{restored}}$ are the gray levels in the original and restored images. NCC is strictly within the range −1∼1. For two identical images, the NCC equals one. Larger NCC means better resemblance of the original and restored images. Figure 2e gave the distributions of NCCs, most of which were larger than 0.99 indicating good representability of the latent features. In this manner, the original V×H diffraction patterns of 1024 × 1024 pixels were reduced to a $N_{f}\times VH$ data matrix (V=64, H=146, $N_{f}=4096$), and denoted as M hereinafter.

Further reduction of $N_{f}$ would certainly deteriorate the restored image. At this stage, data reduction via factorization of the data matrix M would be more effective in preserving the integrity of information. One of the most frequently used factorization schemes is principal component analysis (PCA) which transforms the latent features of the diffraction patterns into the so-called principal components (PCs) such that the cross-correlations of PCs are zeros. The PCs are customarily ordered according to their variances. In this regard, the whole data set can be compressed without too much loss of the information by discarding the PCs that exhibit minimum variances.

Another candidate for factorization is non-negative matrix factorization (NMF) which has been used in processing electron microscopy images [35,38]. In our practice, we found that NMF costed much more computation time than PCA. Meanwhile, NMF had the risk of converging at a local minimum rather than the global minimum. Therefore, NMF is not considered in this work.

The PCA is in essence the singular value decomposition (SVD) of the data matrix M:

(2) $$\begin{array}{c} \;M=U\Lambda V^{T}.\# \end{array}$$

Λ is a $N_{f}\times N_{f}$ diagonal matrix consisting of the square roots of the eigenvalues of the covariance matrix $MM^{T}$, with the PCs stored in the columns of the $HW\times N_{f}$ orthonormal matrix V. The diagonal elements of $\Lambda^{2}$ are in proportion to the variances of their corresponding PCs.

**Figure 3:**
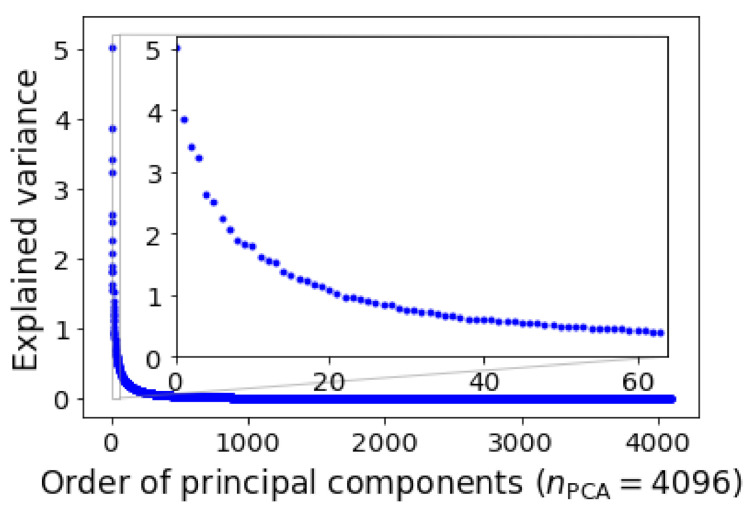
Explained variance of principal components. The first 64 largest explained variances are highlighted in the inset.

We applied the PCA to the data matrix M with Scikit-Learn [37], and displayed the variances of PCs in descending order in Figure 3, in which the variances of the first 64 PCs were shown in the inset. The distribution was highly skewed but the skewness was not as significant as in the work of Song et al. [31], therefore more PCs needed to be truncated.

To sum up, the pipeline for feature extraction consisted of (i) pixel agglomeration with HAC algorithm and subsequently (ii) factorization of data matrix via PCA. It should be noted that neither of the step was indispensable. The design of the pipeline was a compromise between the computational capacity and the integrity of the information. Indeed, although PCA could reduce the size of data with less loss of information, it was computationally more expensive than pixel agglomeration by HAC; in this regard, HAC algorithm should be implemented in the first place to reduce the data to a size handleable by PCA.

### 3.2. Grid Segmentation

After extracting $N_{\mathrm{PCA}}$ features from each diffraction pattern, the scanning grid was ready to be segmented by merging the samples with “similar” features. In the stage of clustering, we used the HAC algorithm again. The most significant advantage of HAC algorithm over other clustering algorithms, e.g., K-Means, affinity propagation, etc., is that the connectivity of the adjacent grid points can be exploited to ensure the continuities of the segmentations. The computation time can also be significantly reduced since only the adjacent grid points shall be considered to merge.

HAC algorithms can operate in two modes: (i) by specifying the number of clusters; (ii) by specifying the distance threshold above which the grid points would not be merged, and the number of clusters will be determined automatically. In the stage of feature extraction, we used the first mode, and here we used the second mode for the segmentation of grid. The rest of the setting was the same as in feature extraction and not recapitulated herein.

**Figure 4:**
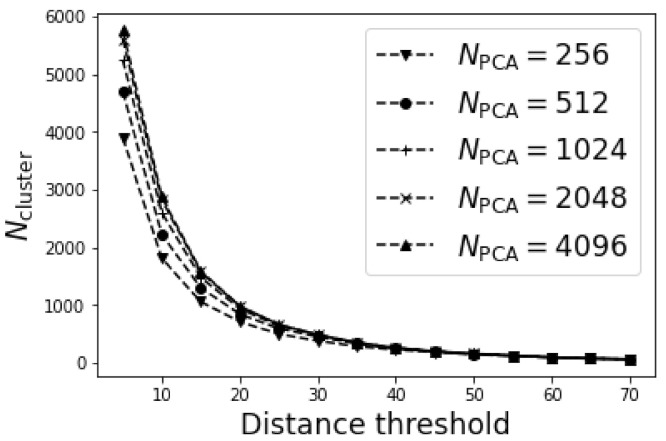
The variation of number of clusters $N_{\mathrm{cluster}}$ with the distance threshold.

Figure 4 plotted the variation of determined number of clusters $N_{\mathrm{cluster}}$ with distance threshold for different $N_{\mathrm{PCA}}$. It was not surprising that the determined $N_{\mathrm{cluster}}$ decreased monotonically with the distance threshold since the adjacent grid points were more likely to be merged under larger distance threshold. The determined $N_{\mathrm{cluster}}$ increased marginally with $N_{\mathrm{PCA}}$ for $N_{\mathrm{PCA}}\geq 1024$, suggesting a good representability of 1024 PCs. Therefore, to save computation time, we adopted $N_{\mathrm{PCA}}=1024$ in the following discussion.

**Figure 5:**
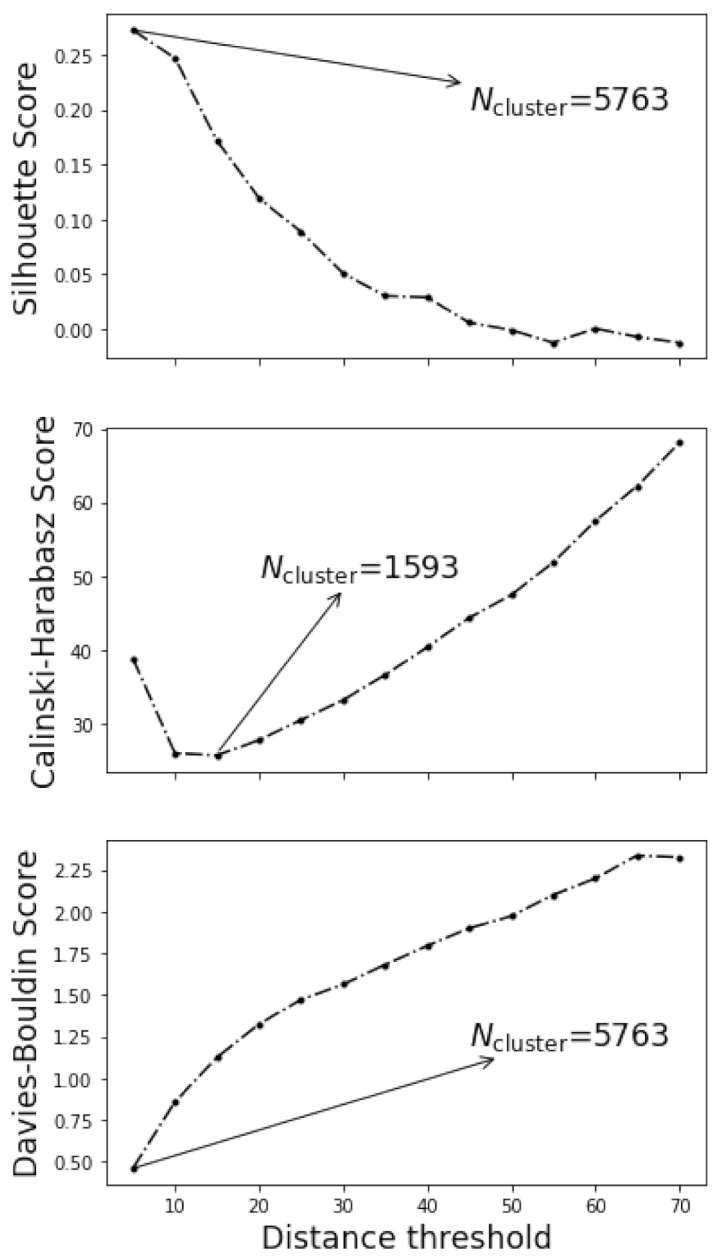
The variation of the silhouette scores, Calinski-Harabasz scores, and Davies-Bouldin scores with the threshold distances. The number of cluster determined from each indices was arrowed.

Indeed, the choice of distance threshold was rather subjective. In two extreme cases, too small distance threshold might lead to the maximum of VH clusters in which individual grid point was considered to be one cluster, whilst too large distance threshold might lead to a single cluster incorporating all grid points. The appropriateness of the clustering is customarily measured by the silhouette score [39], Calinski-Harabasz score [40], and Davies-Bouldin score [41] of the clustering. Silhouette score is positively related to the goodness of the clustering, while the Calinski-Harabasz score and Davies-Bouldin score are the opposite. Figure 5 plotted the variation of the silhouette scores, Calinski-Harabasz scores, and Davies-Bouldin scores with the threshold distances, along with the determined optimum number of clusters arrowed in each subfigure. Both silhouette scores and Davies-Bouldin scores implied that the smaller the distance threshold the better the clustering, while a minimum of 1593 clusters could be observed in Calinski-Harabasz scores.

**Figure 6:**
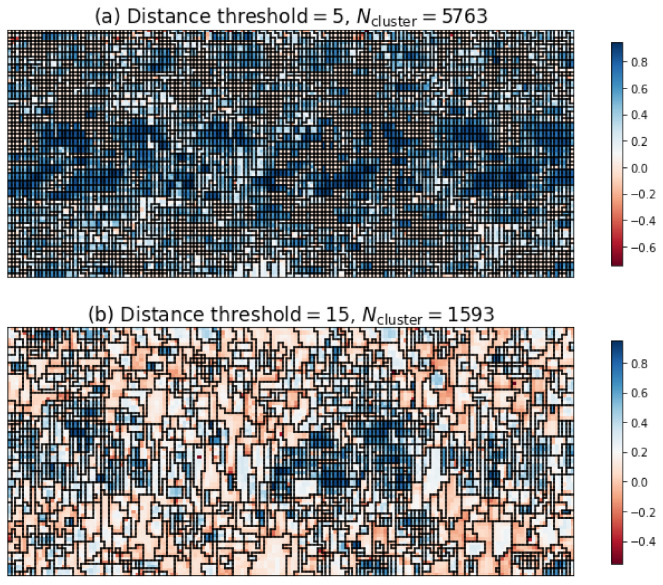
(**a**) the optimal segmentation judging from the silhouette scores and Davies-Bouldin scores; (**b**) the optimal segmentation judging from the Calinski-Harabasz scores. The coloring is given by the silhouette coefficients. The fatigue loading is along the vertical direction. The grid points were spaced by 2 μm vertically and horizontally.

The three indices have not reached a consensus on the optimum number of clusters. Figure 6a plots the optimal segmentation judging from the silhouette scores and Davies-Bouldin scores, and Figure 6b the optimal segmentation judging from the Calinski-Harabasz scores. The coloring of Figure 6 reflected the silhouette coefficient distribution [39]. For individual grid point, the silhouette coefficient is a metric of the suitability of its clustering, and defined as:

(3) $$\begin{array}{c} s=\frac{b-a}{\max\left(a,b\right)}, \end{array}$$

where a and b are the mean intra-cluster distance and the mean nearest-cluster distance for the UA. Silhouette coefficient ranges between −1~1. A low value suggests the ambiguity of grid point labeling. Value near zero indicates overlapping clusters. The silhouette score in Figure 5 is the average of the silhouette coefficients of all grid points.

Both figures were mosaic, especially Figure 6a. After all, after the fatigue loading complex dislocation structures grew and gave rise to the intragranular misorientation. Neighboring lattices slightly disoriented might be treated as different grains. Therefore, the determination of optimal distance threshold from the three indices was of little use in dealing with the fatigued samples.

Herein, we presented a statistics-oriented criterion to decide the optimal distance threshold. In the community of material scientists, the grain size distribution (volume, intersected area or intercept length) in a polycrystal has long been recognized as lognormal as a rule of thumb [42]. Recently, Tang et al. [43] generalized a universal law that the local strain at subgrain scale should obey lognormal distribution irrespective of deformation mechanism (dislocation slip, phase transformation, or twinning) and phase content of the polycrystal. Since the development of grain substructure is related to the lognormally distributed local strain, the lognormality of the substructure size distribution was likely to be inherited after fatigue loading. The reasoning above led us to examine the goodness of clustering from the statistics of the substructure sizes.

Denoting the number of grid points in the *i*th cluster as $A_{i}$, the lognormality of the cluster size distribution was examined by the Kolmogorov-Smirnov (KS) test with the following steps:

(1) Calculate the logarithms of the number of grid points $\log A_{i}$, and the mean μ and standard deviation σ of $\log A_{i}$s;
(2) Normalize $\log A_{i}$ into $X_{i}=\left(\log A_{i}-\mu \right)/\sigma$. Calculate the empirical cumulative distribution function of $X_{i}$, i.e., $G\left(X\right)=n\left(X\right)/N$, where $n\left(X\right)$ is the number of $X_{i}$s smaller than X and N is the total number of clusters;
(3) Calculate the maximum absolute difference between the empirical cumulative distribution function $G\left(X\right)$ and the theoretical cumulative distribution function of standard normal distribution $F\left(X\right)$, i.e., $D=\sup\left|F\left(x\right)-G\left(x\right)\right|$. The distance will be termed as KS distance hereinafter.

**Figure 7:**
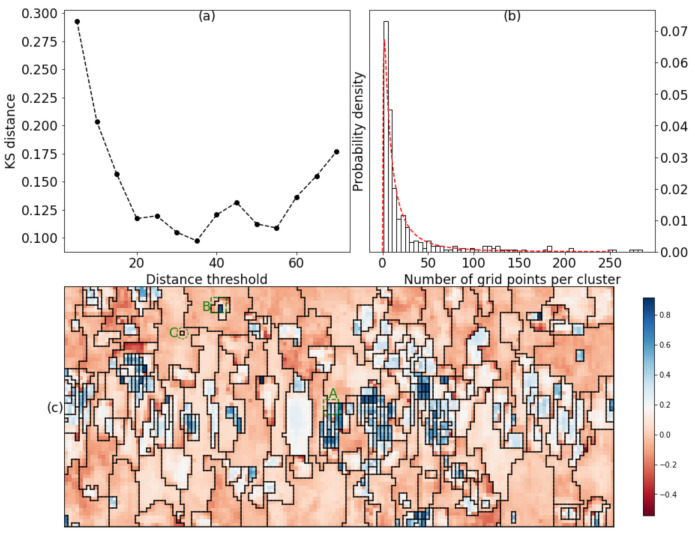
(**a**) the variation of KS distances with the distance threshold; (**b**) the histogram of the grid points per cluster along with the theoretical lognormal probability distribution curve; (**c**) the optimum segmentation result decided from the proposed criterion, the color map is given by the silhouette coefficient distribution, the fatigue loading is along the vertical direction. The grid points were spaced by 2 μm vertically and horizontally.

In this sense, smaller KS distance indicates better lognormality of the distribution. Figure 7a presents the variation of KS distances with the distance threshold, in which the minimum of KS distances occurred at distance threshold equal to 35 and the determined number of clusters was 347. Figure 7b shows the histogram of the grid points per cluster along with the theoretical lognormal probability distribution curve. The curve fitted nicely with the histogram.

## 4. Discussion

Figure 7c displayed the optimum segmentation result decided from the proposed criterion along with the mapping of the silhouette coefficients. The originally equiaxed grain structured became elongated after the fatigue loading. To examine the validity of the clustering, we checked the diffraction images of subgrids A and B (enclosed by green 4 × 4 squares respectively in Figure 7c).

**Figure 8:**
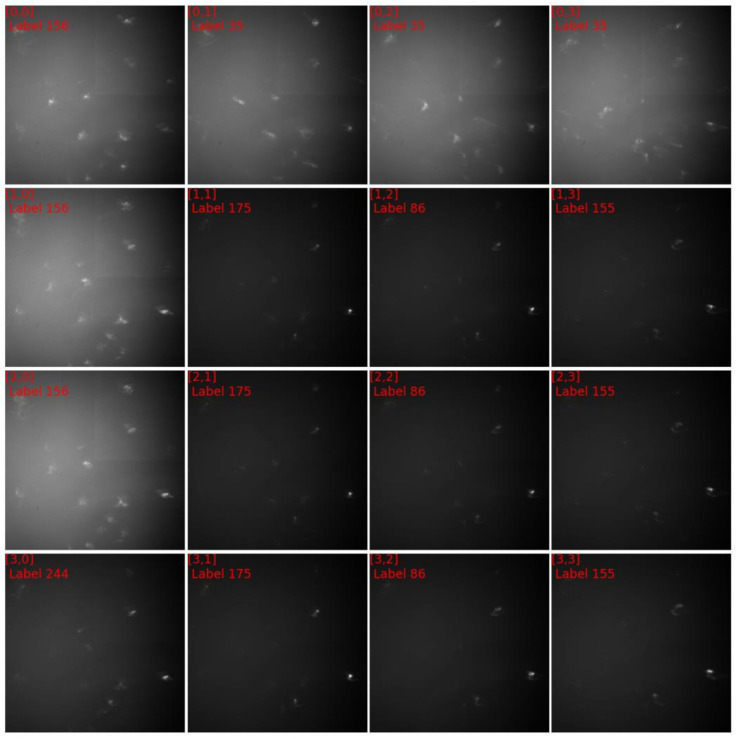
The diffraction images of subgrid A in Figure 7c.

**Figure 9:**
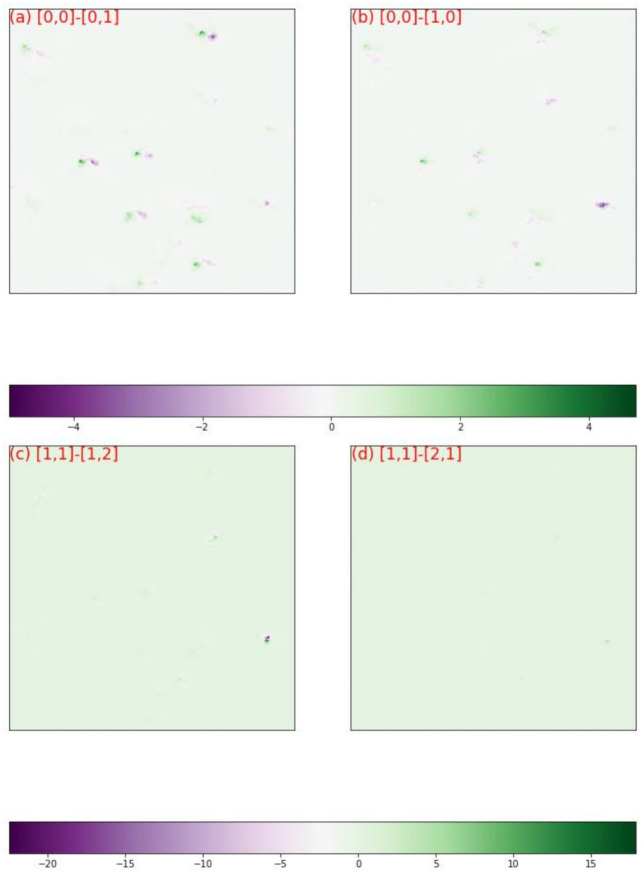
The discrepancy map between the normalized images in Figure 8, (**a**) between [0,0] and [0,1]; (**b**) between [0,0] and [1,0]; (**c**) between [1,1] and [1,2]; (**d**) between [1,1] and [2,1].

Figure 8 showed the diffraction patterns of subgrid A, in which the grid coordinates along with their numbers of clusters were labeled. The diffraction patterns of cluster 156 were distinctively different from those of cluster 175 and those of cluster 244. The diffraction patterns of cluster 35 appeared homologue with those of cluster 156, therefore a quantitative comparison was needed to visualize their discrepancies. Figure 9a,b plotted respectively the discrepancies between the normalized diffraction patterns of [0,0] and [0,1], which belonged to different clusters, and the discrepancies between the normalized diffraction patterns of [0,0] and [0,1], which belonged to one cluster. Obviously, the diffraction pattern of [0,0] was more similar to that of [1,0] than that of [0,1], validating the segmentation that grouped [0,0] and [1,0] in one cluster. The same analysis was applied to the diffraction patterns [1,1], [2,1] and [1,2], and validated the segmentation results (Figure 9c,d). The relative displacements of diffraction spots among the homologue diffraction patterns could be measured precisely with DIC technique to reveal their lattice misorientation [44,45].

**Figure 10:**
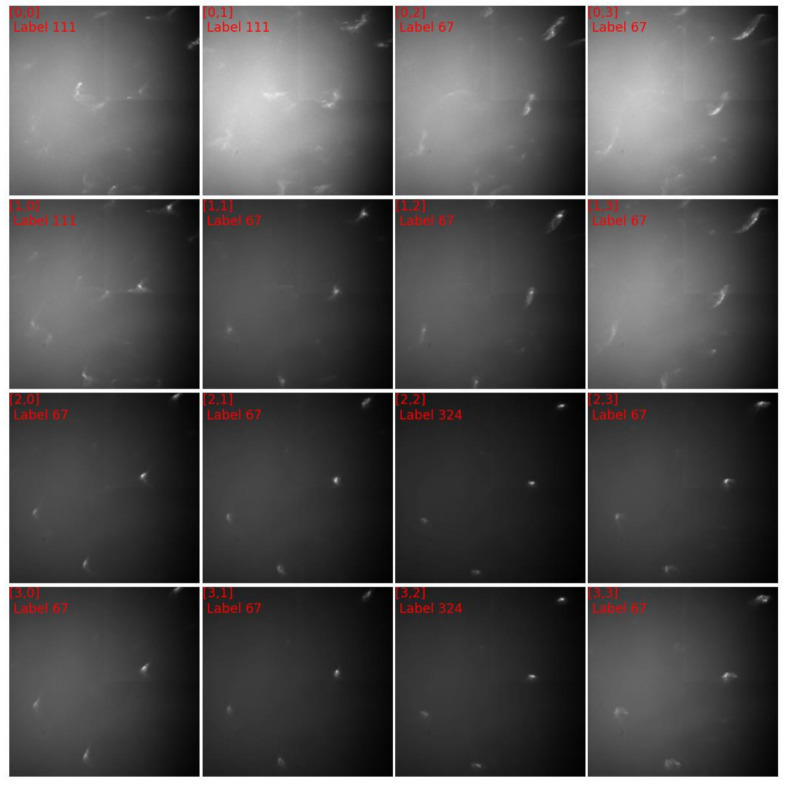
The diffraction images of subgrid B in Figure 7c.

The diffraction patterns of subgrid B in Figure 10 shared some sort of homology suggesting that cluster 67, 111, and 324 originated from a single grain. The diffraction spots in the three clusters differed in the degrees of blurring:

(1) in cluster 324, the spots were slightly streaked due to the penetration of the X-ray; the distinctively high silhouette coefficients indicated that the diffraction patterns in cluster 324 shared high resemblance compared to patterns in other clusters.
(2) in cluster 67, the spots were elongated unidirectionally implying that one slip system was predominantly activated; the more distant from cluster 324 the more blurred the spots.
(3) in cluster 111, some spots were multidirectionally streaked implying the activation of multiple slip systems; some spots were highly blurred or even indiscernable suggesting innegligible amount of statistically stored dislocations (SSDs) in the illuminated volume.

The progressively blurring of spots from the interior (cluster 324) to the border (cluster 111) of the grain conveyed a picture of dislocation pile-up in which the mobile dislocations became sessile at the grain boundaries and obstructed the dislocation emitted from the interior of grain. The pile-up of primary dislocations led to the activation of other slip systems, resulting in multidirectionally streaked spots (e.g., diffraction pattern [1,0] in Figure 10).

The subgrids A and B were two typical examples of the substructure development in response to the constrain imposed by their neighboring region. Their varying streaked diffraction spots deserved further depth-resolved characterization via DAXM, either in polychromatic mode [16] or in scanning monochromatic mode [46], to have a comprehensive understanding of the dislocation substructure in fatigued polycrystalline sample.

**Figure 11:**
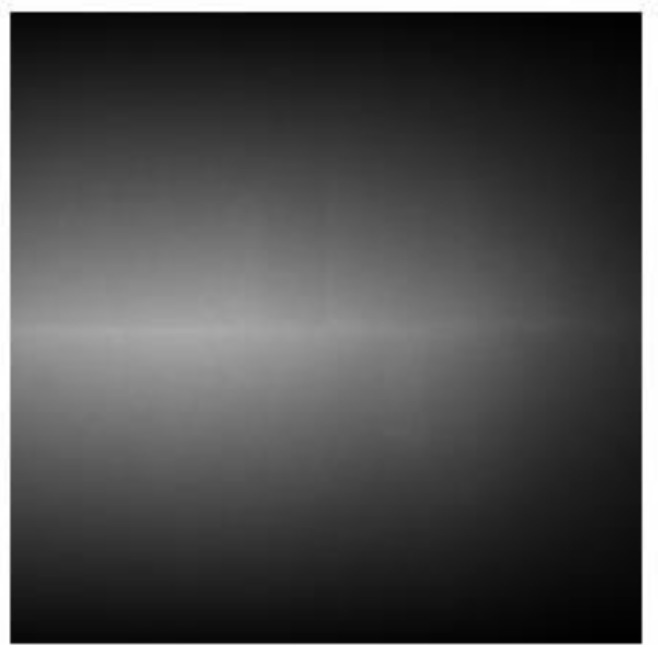
The diffraction images of subgrid C in Figure 7c.

We also noticed some clusters that contained only one grid point, for example, the subgrid C encircled in Figure 7c. The diffraction image of the subgrid C was shown in Figure 11 wherein the image seemed saturated by the background noise and no spot was seen. This was probably due to the artefacts in the scanning.

## 5. Summary

In the present work, an indexation-free treatment of raster scanning Laue microdiffraction patterns was proposed to have a fast segmentation of the scanning grids based on their underlying diffraction patterns, whereas the conventional treatment had to index diffraction patterns one-by-one and thus could hardly give on-the-fly feedback. The final segmentation was informative of the underlying microstructure from which several regions deserving further investigation could be identified.

In analogy to the preceding work [31,32,35,38], the treatment consisted of two steps: (i) feature extraction and (ii) grid segmentation. For pragmatic consideration, we adopted unsupervised machine learning algorithms since the training data might not be always available. The proposed protocol differed from the preceding work from the following aspect:

(1) Both steps used the HAC algorithm since HAC algorithm could exploit the 2D connectivity in both the pixels of the diffraction patterns and the grid points of the raster scanning to ensure the continuity of segments and save the computation time.
(2) A statistics-oriented criterion was proposed as a guideline to set the distance threshold in HAC algorithm which determined the number of segmentations, which yielded at least in our case more realist results than conventional criterion.

The application of machine learning to raster scanning diffraction images is a case-by-case issue. The proposed protocol, which was useful in our case, has the potential to be applied in other cases, and help economize the limited beamtime.

## Data Availability

Data available in a publicly accessible repository.
